# Comment on “Regulation of immunity during visceral *Leishmania* infection” and further discussions about the role of antibodies in infections with *Leishmania*

**DOI:** 10.1186/s13071-016-1669-0

**Published:** 2016-07-07

**Authors:** Luiz Gustavo Gardinassi, Isabel Kinney Ferreira de Miranda Santos

**Affiliations:** Departamento de Bioquímica e Imunologia, Faculdade de Medicina de Ribeirão Preto, Universidade de São Paulo, Ribeirão Preto, São Paulo Brazil

**Keywords:** Leishmaniasis, Antibodies, Fc receptors, Fc N-glycosylation, Immunopathology, Protection

## Abstract

Comments on the article “Regulation of immunity during visceral *Leishmania* infection” published in *Parasites & Vectors* 2016, 9:118, and further discussions about the role of antibodies in infections with *Leishmania*.

## Letter to the editor

Rodrigues and colleagues recently presented in this journal a comprehensive and timely review of the current state of knowledge about immunity to visceralizing species of *Leishmania* in humans and in animal models [1]. We read with particular interest the topic they devoted to studies that evaluated the role of antibodies, B cells and T follicular helper cells during infections with these intracellular parasites. We strongly agree with the authors’ conclusions about the need to reassess the role of antibodies in these infections, wherein they state that *“Rather than considering the role of antibodies solely as pathological or irrelevant, it is perhaps wiser to acknowledge that these molecules may play both protective and non-protective roles during VL”*. However, we would like to add further information from studies that provide important evidence and concepts that also support a crucial role of antibodies during infections with *Leishmania* and that were not considered in their review.

As pointed out by Arturo Casadevall, the rationale for dichotomizing types of immunity against extracellular and intracellular pathogens into antibody-mediated and cell-mediated, respectively, is based on concepts that probably originated in the great debate between scientists advocating humoral or cellular immunity at the beginning of the 20th century [2]. However, this concept cannot be applied to every known pathogen, whereby antibodies have been shown to protect against at least some intracellular pathogens such as *Trypanosoma cruzi* [3, 4], *Histoplasma capsulatum* [5] and *Mycobacterium tuberculosis* [6]. Since the majority of early studies on the leishmaniases did not correlate antibody-mediated immunity with protection, many questions about the role of B lymphocytes and antibodies during such infections remained unanswered. However, an initial proof of concept that antibodies can be important for controlling infections with *Leishmania* comes from the observation that pathogenic Trypanosomatidae express receptors for IgG Fc [7, 8] or proteases for IgG [9], possibly to escape from this effector mechanism.

In the early 80’s Anderson and colleagues [10] demonstrated that the protective effect of a monoclonal antibody raised against *L. mexicana* was dependent on the number of antibody-opsonized parasites inoculated into BALB/c mice [10]. Furthermore, another study showed that monoclonal antibodies raised against *L. infantum* conferred passive cross-protection against infections with *L. amazonensis* and *L. major* in mice [11]. As addressed by Rodrigues and colleagues [1], a study demonstrated that protective immunity against *L. major* required efficient uptake of IgG-opsonized parasites by dendritic cells through Fc-gamma receptors (FcγR) I and III [12]. More recent work revealed that elimination of *L. amazonensis* depends in part on the FcγR common-chain and NADPH oxidase-generated superoxide from infected macrophages *in vitro* [13]. In contrast, a study showed that interactions of mouse IgG1 with FcγRIII is detrimental in infections with *L. mexicana,* but not interactions with IgG2a/c or IgG3 [14]. Together, these data suggest that the signaling pathways through which phagocytes are activated might be fundamental for the functional profile that these cells will acquire and, therefore, for their effects on polarizing the cell populations that will comprise *Leishmania*-specific acquired immunity.

Notably, mice that lack immunoglobulin-bearing B cells (JhD BALB/c) are considered to be relatively resistant to infection with *L. pifanoi, L. amazonensis* and *L. infantum* and passive transfer of B cells, immune serum or purified antibodies to chronically infected JhD BALB/c mice restored susceptibility to infection, respectively [15–17]. In addition, μMT mice (assumed to lack mature B cells) also exhibit a relative resistance when infected with *L. major* LV39 (BALB/c) [18] or *L. donovani* (C57BL/6) [19]; however, other work indicates that μ-chain-deficient mice possess functional B-1 cells and can produce non-specific IgG [20, 21]. Indeed, the studies mentioned above did not discriminate between subsets of cells derived from B lymphocytes such as plasma cells, regulatory B cells or marginal zone B cells, which exhibit different functions depending on the context of infection with *Leishmania* [22–28]. We also highlight that, while mice (BALB/c or C57BL/6) are acutely susceptible to these parasites, they do not develop chronic and progressive disease after infection with *L. donovani* or *L. infantum* [29]. Therefore, the role of antibodies in these mouse models of visceral leishmaniasis (VL) and its translation into immunopathological features observed in progressive disease as seen in humans and non-human primates, dogs and hamsters, may warrant reinterpretation. Accordingly, Reis and colleagues [30] observed a strong correlation between levels of IgG1 antibodies specific for soluble antigens from *L. infantum* promastigotes and an asymptomatic outcome of infection and also a correlation between IgG2 of the same specificity and manifestation of disease in dogs. In another study, Oliveira and colleagues [31] observed that dogs presenting with asymptomatic infections with *L. infantum* have significantly lower levels of IgG2 antibodies specific for a crude antigen extract from these parasites than symptomatic dogs. Interestingly, these authors also observed that dogs vaccinated with Leishimmune® (Fort Dodge Animal Health) and supposedly protected against disease, present negligible levels of IgG1, IgG3 e IgG4 antibodies and high levels of IgG2 antibodies against this crude antigen extract.

As pointed out by Rodrigues and colleagues in their review [1], the dose of parasites may influence the resulting immune response [32]. Menon & Bretscher [33] also determined that the dose of *Leishmania* affects the outcome of infection even in genetically susceptible hosts: BALB/c mice can control infection with *L. major*, a visceralizing parasite in this host, if they receive a low dose of parasites. Dominguez and colleagues [34] and Moreno and colleagues [35] showed that natural antibodies present in normal human sera rapidly (i.e. in 2.5 min) cause lysis of 85–95 % of infective metacyclic *Leishmania* promastigotes by means of the classical pathway of complement. Thus, *Leishmania*-reactive antibodies presenting an adequate profile may result in manageable doses of parasites at crucial initial stages of infection with *Leishmania* by promoting lysis of infective promastigotes inoculated by vectors. This mechanism does not exclude a further role for antibodies with adequate profiles in activating microbicidal mechanisms and expansion of efficacious effector responses through appropriate signaling pathways in *Leishmania*-infected phagocytic and antigen-presenting cells.

Finally, we wish to point out that not only variability at the level of protein within IgG subclasses and the different FcR affects effector mechanisms of antibody-mediated immunity: interactions of IgGs with type I FcR (the canonical FcγRI, FcγRIIa, FcγRIIb, FcγRIIc, FcγRIIIa, FcγRIIIb) or type II FcR (DC-SIGN and CD23) [36]; or with mannan-binding lectin [37] and the complement component C1q [38] are also regulated by the patterns of N-linked, biantennary glycan structures attached to Asn 297 of the IgG heavy chain [39]. In a recent study, we and our colleagues applied a robust method of IgG Fc N-glycopeptide profiling that demonstrated profound alterations in the patterns of IgG Fc N-glycosylation of VL patients compared with those of asymptomatic individuals and non-infected controls [40]. In fact, the profile of IgG Fc glycans presented by the VL patients that we examined is compatible with and may contribute to the imbalance of the inflammatory milieu that Rodrigues and colleagues suggest is unfavorable for proper differentiation of T follicular helper cells [1]. In our opinion, those alterations are extremely relevant and should be considered in future studies addressing immunopathological or protective responses in infections with *Leishmania*, which might depend on the clinical context after infection and possibly affect bystander inflammatory processes [41], B cell activation and regulation [42] and antibody-mediated effector functions.

## Abbreviations

FcγR, Fc-gamma receptor; VL, visceral leishmaniasis
